# ‘Surrounding yourself with beauty’: exploring the health promotion potential of a rural garden appreciation group

**DOI:** 10.1093/heapro/daad010

**Published:** 2023-02-25

**Authors:** Leith Symes, Nyssa Hadgraft, Pauline Marsh, Sonia Nuttman, Jonathan Kingsley

**Affiliations:** School of Health Sciences, Swinburne University of Technology, 12 Wakefield Street (Swinburne Place West), Hawthorn, Victoria 3122, Australia; Centre of Urban Transitions, Swinburne University of Technology, Level 1 EW Building, Hawthorn, Victoria 3122, Australia; Baker Heart and Diabetes Institute, 75 Commercial Rd, Melbourne, Victoria 3004, Australia; Wicking Dementia Research and Education Centre, College of Health and Medicine, University of Tasmania, Liverpool St, Hobart, Tasmania 7001, Australia; School of Health and Social Development, Deakin University, 221 Burwood Hwy, Burwood Victoria 3125, Australia; School of Health Sciences, Swinburne University of Technology, 12 Wakefield Street (Swinburne Place West), Hawthorn, Victoria 3122, Australia; Centre of Urban Transitions, Swinburne University of Technology, Level 1 EW Building, Hawthorn, Victoria 3122, Australia

**Keywords:** garden, rural, well-being, social connection

## Abstract

Gardening has the potential to enhance health and well-being, through increased physical activity and social connectedness. However, while much is known about the benefits of garden activities, less is known about the potential health implications of more passive forms of engagement with gardens, for example, viewing gardens. In addition, much garden research is undertaken in urban settings, leaving little known about potential health impacts for rural populations. The present study explored these research gaps by gaining an understanding of the experiences and perspectives of members of a gardening appreciation group in rural Australia: The Colac Horticultural and Marvellous Property Appreciation Society (CHAMPAS). A phenomenological, qualitative methodology was applied, using semi-structured interviews for data collection. Eleven participants were selected using purposive and snowball sampling and the data were analysed by applying interpretive, reflexive thematic analysis. Four main themes and supporting sub-themes were generated. The four main themes were: (i) motivations for maintaining participation in CHAMPAS; (ii) social connections and friendships, formed from membership; (iii) sense of community and structure of CHAMPAS and (iv) the perceived health and well-being benefits of continued involvement in this group. This study found that members perceived health and well-being benefits stemmed from CHAMPAS facilitation of social connectedness, function as a community group and a way for members to share a love for home gardening. This study provides insights into the perceived and potential health-promoting effects of garden appreciation groups for rural populations.

## INTRODUCTION

A raft of evidence now exists that confirms gardens and gardening are a way to access and engage in green spaces for significant health and well-being benefits (van den Berg et al., 2010; Cervinka et al., 2016; Brindley et al., 2018; Chalmin-Pui et al., 2021). One cross-sectional study demonstrates that adults over 62 years of age involved in allotment gardening performed better on all measures of health than those who were not involved in gardening (van den Berg et al., 2010). Thus, it could be assumed that older adults gain greater benefits from involvement in gardening activities.

Allotment and community gardening, in particular, has been shown to have a broad range of health and well-being benefits for participants (Kingsley et al., 2009). Community gardening has been shown to allow for connections to be formed among people who share similar values and to lead to the breaking down of cultural barriers, which builds more socially sustainable communities and facilitates social connections and functions (Christensen, 2017; Kingsley et al., 2020). Enjoyment and social benefits are often stated as the primary motivation for people’s participation in community gardening, rather than for reasons of physical health (Kingsley et al., 2019; Chalmin-Pui et al., 2021). This raises the question of whether the same can be said of groups and/or social networks that meet to appreciate gardens rather than practice gardening. Lewi et al. (2014) note that touring gardens allows for a rewarding poetic and personal exchange among people and historical landscapes. Other research has indicated that there are particular well-being and quality of life benefits of gardening as people age (Fielder and Marsh, 2020). Roger Ulrich’s (1984) now famous study of improved rates of recovery from surgery for those who had a view of nature from a window would also indicate a strong possibility. While not involving the physical aspect of gardening, gardening appreciation groups may provide health and well-being benefits through their ability to foster social connectedness among individuals with shared interests and facilitating participants spending more time ‘viewing’ green spaces. We define garden appreciation groups as:

(i) Communities of people who share accepted elements of gardening definitions, including viewing gardens and having strong associations with the development of community and social connections (Okvat and Zautra, 2011; Ernwein, 2014; Howarth et al., 2020); and,
(ii) Having the inherent esthetic motivations of being involved in natural and green spaces (Wilson, 1984; Kellert, 1996).

### Context of the present study

Due to significant increases in global populations residing in urban areas (World Health Organisation, 2021), it is of no surprise that much of the recent research into gardening is urban-focused, meaning less is known about the health promotion potential of gardening and garden appreciation activities in rural areas (Kingsley et al., 2019; Kendal et al., 2020). It might be assumed that due to greater geographical proximity to nature, people living outside of cities have stronger nature connections. However, agricultural landscapes, which typically dominate rural areas in Australia, have been found to negatively affect human–nature connectedness (Holmes, 2006; Seymour, 2016; Binks et al., 2018; Riechers et al., 2021). Agricultural landscapes facilitate landscape simplification which negatively influences social relations, social cohesion and cultural identity (Riechers et al., 2021). About 8 million Australians live outside of major cities and their surrounds which equates to 32.5% of the total population (Australian Bureau of Statistics [ABS], 2019). Rural and remote Australians experience poorer health and well-being, in part, due to impacts associated with social determinants of health (Australian Institute of Health and Welfare [AIHW], 2018, 2019). Lower life expectancy, higher rates of suicide and higher rates of chronic diseases amongst rural and remote populations are just a few examples (AIHW, 2018, 2019; Health Direct, 2021).

Given these health disparities and related risk factors, health promotion efforts that encourage people to spend more time in green spaces and also promote social connectedness may be beneficial for improving health and well-being in rural populations. The present study, therefore, sought to explore the experiences of members involved in a rural garden appreciation group. The specific aim was to understand the perceived health, well-being, and social connectedness implications of membership in this group by asking two research questions: (i) what motivates members to participate in the group; and (ii) how is participation in the group perceived to influence health and well-being?

## METHODOLOGY

### Study site

Colac Horticultural and Marvellous Properties Appreciation Society Incorporated (CHAMPAS) is a not-for-profit organisation that was established in 2017 to provide an avenue for the appreciation of gardens and how they complement homes of historical or notable significance (CHAMPAS, 2017). Although it is recognised that this group both appreciates gardening and historical/significant homes, the focus of this group (after consultation with the president and committee members of CHAMPAS) was gardens predominantly. The organisation operates with a formal governance committee and holds monthly face-to-face events, including (mainly) outings to local properties with the occasional long-distance outing for garden and home tours and infrequent presentations by horticultural experts (CHAMPAS, 2017). These outings often include the provision and sharing of refreshments and socialising. CHAMPAS has a fluctuating membership of approximately 50 people. Some members kept in touch between meetings, but this was not always the case. However, at the time of this study, the frequency of events and member engagement was reduced due to the COVID-19 pandemic.

CHAMPAS is based in the rural town of Colac in the Australian state of Victoria which has a population of 12,584 (ABS, 2021). Colac itself includes a range of commercial and residential zones, but notably, most of the land surrounding Colac is purposed for agricultural use (VICPLAN, 2021).

### Research methods

The study utilised a phenomenological qualitative approach to support our aim of understanding participants’ experiences of being involved in a gardening appreciation group. This research approach acknowledges the value of learning from people’s experiences, through the elicitation of descriptive data that comes from the people involved in the particular phenomenon (Creswell, 2014). Phenomenology privileges an understanding of individual experiences as relational, that is, as intersubjective—between each other and the world around us (Creswell and Miller, 2000; Flood, 2010; Coleman 2016) and seeks to understand and generate meanings of these experiences (Neubauer et al., 2019). The philosophical underpinning of our approach is interpretive: we pay attention to everyday experiences that participants offer, acknowledge our own subjectivity, and reflect on both the participants and our own relationships to the phenomenon when interpreting the data (Coleman, 2016; Neubauer et al., 2019). A personal relationship between the lead author and the CHAMPAS group was already established prior to the research project. Being an ‘insider’ researcher is not only complementary to reflexive interpretive design but also beneficial for qualitative approaches (Dwyer and Buckle, 2009; Ross, 2017). Ethics was approved for this project by the Swinburne University of Technology Human Research Ethics Committee [Ethics project ID: 5589]. Early in the research project, we recognised the ethical concerns associated with naming an organisation like CHAMPAS so we got consent from the president and committee to use their name in this ethics application. After completing this initial manuscript, we also followed this question up again to know if we could still use their name. The president and committee were still supportive of CHAMPAS being named in this publication.

### Semi-structured interviews

Semi-structured interviews were utilised as a method as it allowed the generation of information-rich material to gain an understanding of the experiences of CHAMPAS members. This was done via a range of questions that were developed to facilitate conversation between the interviewer and participant to reveal experiences and insights not known to the researcher (Tracy, 2010). The semi-structured interview consisted of 18 questions about the participants’ health, well-being, and their interactions with CHAMPAS and the broader community (examples in Table 1: For full list of questions please see Supplementary file). These interviews took place via Microsoft Teams due to COVID-19 restrictions in place at the time.

### Data collection

CHAMPAS was purposively selected for this study as its members were information-rich sources due to their direct experience of being involved in a rural garden appreciation group. This approach is consistent with Robinson’s (2014) strategy of purposive sampling which outlines the importance of the researcher’s awareness of potentially valuable, unique, and important perspectives. The selection process was facilitated through a gatekeeper at CHAMPAS. Contact was initially established with participants through the CHAMPAS president who passed on the recruiting material to members. The president contacted prospective participants, as part of the group’s regular email newsletter. Prospective participants contacted the lead author, expressing their interest to participate, and provided informed consent prior to interviews taking place. To assist the recruitment process, snowball sampling was employed with participants being invited to suggest other members of CHAMPAS who may wish to participate. This method of recruitment was used to maximise potential participants (Robinson, 2014).

Inclusion criteria were framed around member involvement in CHAMPAS for at least one year and being at least 18 years of age. Interviews were approximately 30 min each and were recorded and transcribed verbatim. The data collected were edited to remove identifying features and all names were changed to pseudonyms. Fourteen days after the interview, the participants were offered an opportunity to review their transcripts (none took up this offer).

### Data analysis

Data were analysed using an interpretive, reflexive thematic approach, that is, a non-linear process of reading, coding, reviewing, and discussing (Braun and Clarke, 2019). Coding was managed by the lead author and supported by dialogue and feedback from the supporting researchers. The transcripts of each interview were read through several times by the lead author and codes were assigned and relevant quotes were pulled from the transcript. Each time a code was assigned to the quote, a comment was noted describing the researcher’s perspective of its significance and meaning to the research questions. This was a dynamic process with codes and themes being included, adapted, omitted, and refined through the process of dialogue and feedback with the research team. Complementing the reflexive thematic process, the lead author kept a journal of notes/emerging themes that occurred as the interviews progressed, to help with reflection and to draw out rich findings. This process generated themes that were ‘stitched’ together to provide a rich narrative, gave trustworthy insights into the experiences of the participants (Braun and Clarke, 2019; Liamputtong, 2020), and enabled the research team to interpret the meaning of their experiences.

## FINDINGS

A total of 11 participants contributed to this study. Of the 11 participants, 1 was male and 10 were female. Participants ages ranged from 45 to 79 years of age. Seven participants were employed either part or full-time, and four were retired.

Four main themes and several supporting sub-themes were generated by the analysis. The main themes were:

(i) Motivations of participation: the factors that made participants want to engage and maintain participation in CHAMPAS.
(ii) Social connections: the new friendships made and how these connections developed.
(iii) Community and structure: explanations of how participants interacted and experienced CHAMPAS, and the broader community of Colac.
(iv) Perceptions of health and well-being: participants’ perceptions of the influence CHAMPAS had on their health and well-being.

In the following section, each of these theme areas will be discussed in more detail and provide supporting example data.

### Motivations of participation

The motivations for participation in CHAMPAS were diverse but had overlapping elements. These ranged from enjoying the ‘different’ experience that CHAMPAS offered, wanting to improve their knowledge of their local area and gardening, needing something to do, and using CHAMPAS to increase their friendships. Rebecca summarised this by saying her motivations included:

Getting to know the area that I live in and making connections... and then there’s usually an historical element [to CHAMPAS events] … and if there’s not an historical element, then there’s a design element and sometimes you’ll get to meet the architect or the landscape designer

#### More than just gardening

Enjoying the experience of visiting both the gardens and accompanying properties was a driving factor for many participants. Donna explained that her main interest in joining was that it ‘was more than just gardening’. Few were only motivated by their passion for gardens. Some members indicated their passion around the properties and gardens was around learning the history and appreciating the area and its homes. Madeline highlighted the historical value that she gained from CHAMPAS when she said:

…Walking through those gardens and just imagining being there in that period in time … it’s amazing. Sometimes I think I was born in the wrong era.

Some people came to CHAMPAS believing that the gardens were the focus, and others, the properties. Rebecca said that ‘it’s not only the gardens that the members are interested in; they’re interested in the homes generally’. James saw this differently stating that ‘while its magnificent, you know, magnificent properties, appreciation. It’s more the gardening side … they’re all gardeners’. Julie added to this:

You’d have rocks in your head to go [to CHAMPAS] if you didn’t like gardens.

There was a frequently expressed desire amongst participants to learn new skills and increase their knowledge of gardening and design. Donna wanted to expand her knowledge and skill base around gardening and thought that members of CHAMPAS could provide her with what she wanted, when talking about new gardening ideas she was learning, she said: ‘It was getting a broader view of what other people had to offer’. James similarly wanted to learn different approaches but from a home and design perspective:

Being able to go to other people’s places and see what they’re doing … so many ideas from it all … so it was a nice reference point I guess.

Another driver for members joining CHAMPAS was their dissatisfaction with the strict guidelines that other community groups may follow and pursued their desire for a group ‘without all the rules’. Members who had recently retired were collectively looking for something to fill their spare time and thought that CHAMPAS might provide that. Members enjoyed the non-restrictive expectations and leisure activity that came from enjoying the ‘freedom’ that CHAMPAS had associated with it. Julie summarised this ‘freedom’ saying:

I value CHAMPAS more because it’s purely voluntary. I mean, I can either go or not go into any particular thing. It’s unstructured in that sense and I go because I like it.

An example of how CHAMPAS was ‘laid back’ was stressed by the participants saying CHAMPAS is pronounced akin to the Australian slang word for champagne, ‘champers’.

### Social connections

Participants noted that CHAMPAS led to new friendships and built connections that increased their support networks. Social connections were the most recognised theme, with all participants reporting this as a reason for their engagement in CHAMPAS. As Rebecca said:

I love meeting new people and talking to people

#### Making new friendships

Kate detailed her experience making new friends through CHAMPAS and felt she had a range of people she now felt comfortable with. She highlighted that she had made an important friendship with two of the members and that she felt she could visit them at any time ‘without any qualms about it’. Donna and Amy both recognised a need for them to strengthen their social circle in retirement. Amy highlighted that:

It was important to me to be able to be involved or connected to a group and accepted by them and maybe from that have some friendships grow out of it.

CHAMPAS was recognised by participants as being the entity that facilitated new friendships. Angela explained that she felt everyone was friendly and that she was not aware of any disagreements between members. Madeline supported this saying she always felt welcome at CHAMPAS events. Julie summarised the collective experiences of the participants when it came to friendships, saying:

It’s changed the mix of people I know, and [it’s] very enjoyable

The social networks formed through CHAMPAS were perceived by participants to be important in providing them with support through increased available skills and resources. Kate raised her belief that ‘people need people’ and the support that comes from that. The only person who appeared to question the support the group provided was Amy, who originally noted that she didn’t feel support from the group but then clarified this by stating:

I don’t think I could say categorically that they wouldn’t support me because I don’t think I’ve had a need, that I’ve sort of felt that I shouldn’t request support.

#### Support leading to learning new skills and reciprocity

The sharing of skills and resources between participants was frequently reported. Jennie provided a range of examples of members from CHAMPAS offering her advice on her garden, providing her with plants and even getting her into contact with tradespeople that helped to maintain her private garden. Kate sought out advice from members, saying that she often enjoyed ‘picking their brains’ and receiving cuttings. She indicated that there are some prominent members that cultivate many plants to share within the group. Bethany pointed out how willing members were to share:

People are very generous with their ideas, and their time and suggestions

Abigail highlighted that there are no obligations around support saying that CHAMPAS ‘doesn’t obligate its members to raise money’. But she indicated that many members are socially involved outside of CHAMPAS, supporting their community:

…Members of the group are involved in yet another group… It also has a social objective, but to fundraise to support disadvantaged students

Abigail went on to outline how the group supported each other in an organisational capacity. She explained how there is never an issue when something needs to be done in the group; there is no shortage of people ‘putting their hands up’. Although many participants did report that the president of CHAMPAS was consistently the main driver of group functioning. The support that CHAMPAS was indicated to provide to participants also extended into the local community, as a few of the participants outlined CHAMPAS’ involvement in helping a local school, as Rebecca said, ‘Get the kids interested in gardening and growing things’, and donating plant cuttings to the school.

### Community and structure

Ideas about community and the CHAMPAS structure were commonly raised by participants. The perceptions were that the community was most beneficial, with some making more neutral observations. The connections that participants had with other local groups also contributed to their perceptions of community.

Many participants recognised the impact CHAMPAS had on their ‘enjoyable’ experience of the broader Colac community and provided insight into a smaller group. Angela highlighted the change that being a member of CHAMPAS had made in her community giving her:

The opportunity to meet and speak to people that I have known existed but haven’t actually known.

A few members highlighted how it reinforced and strengthened their sense of community. Abigail said CHAMPAS helped her recognise how important her community is. Her interactions with CHAMPAS provided her with a more positive outlook on the broader community of Colac that she would not have had without joining the group.

Some of the participants reported that CHAMPAS itself hadn’t altered their involvement or sense of community, suggesting they were already community-minded prior to joining CHAMPAS. James noted:

We’ve always been involved in community things, so that’s just kind of a way of life for us … it hasn’t changed anything

Many participants reported being members of other community groups while also being involved in CHAMPAS. Participants often made comparisons between these groups. Julie had recently moved from a farm to Colac, and she appreciated that CHAMPAS was a group that suited her interests and not her partner’s. Amy mentioned that her involvement in CHAMPAS had made her aware of other similar groups in Colac. She described that many members were involved in multiple gardening and appreciation groups and that it gave her an opportunity to discover and enjoy these groups that she would not have found otherwise.

Julie had a unique perspective, saying that she found CHAMPAS refreshing because in her words, her other groups in Colac and surrounds had ‘old attitudes to things and they’re very sort of traditional and stuck in the mud’. She stated that what kept her motivated in CHAMPAS is that it was:

Something more innovative, something different.

#### Awareness of diversity

Members were aware of ethnic homogeneity in the group. Madeline noted that the members of CHAMPAS are mostly ‘White Australian’ but that the area of Colac has quite a ‘diverse range of nationalities.’ She and other participants stressed that this was not intentional and that if people from other nationalities ‘wanted to be part of it, they would be most welcome’. Rebecca highlighted that there were no barriers to anyone joining, providing examples of members from as far away as Melbourne, concluding:

We don’t have any exclusivity about membership.

One point frequently raised was the stark difference in the number of men and women in the group. Participants had differing views on why this existed. Rebecca believed that men probably were not as interested in gardening as women, especially when it came to retirement, pointing out that there were other organisations in Colac that catered to men.

### Perceived health and well-being benefits

Participants’ perceptions relating to the health effects of CHAMPAS were mostly positive, however, some members had neutral reactions to questioning about whether CHAMPAS impacted their health and well-being. Notably, when asked this question, participants also often spoke of their personal gardens as a source of health and well-being.

The participants did not perceive CHAMPAS to directly improve their physical fitness. For example, Kate was not entirely sure that her involvement had any direct impact on her physical health, although she highlighted indirect benefits. She said that because of CHAMPAS she was: ‘doing more gardening, like, that makes me more physically active’. Some members were aware of their need for fitness and often talked about gardening as a way of providing one of many avenues to exercise.

Most participants in the group reported a strong positive influence of CHAMPAS on their overall well-being. James provided insight into his experience at CHAMPAS events, saying: ‘it’s the same thing out and about in nature, for the day it’s a really good mental health thing’. Other participants noted the benefits of being in a garden or a green space, often sharing their love for spending ‘lots of time’ in the garden and out in nature. Jennie shared her experience of gardening as a stress-relieving activity, saying:

…It’s very cathartic to get your hands in the dirt and to grow things and see them thrive or die, whichever happens.

However, Jennie did go on to raise that she did not perceive CHAMPAS to have a substantial influence on her mental health, explaining that her ‘mental health is quite stable’.

Another pathway through which CHAMPAS appeared to have potential health-promoting benefits was through reinforcing self-esteem and pride. Rebecca highlighted that she felt it was important to present yourself well, and that this included personal spaces, such as the home and importantly garden, saying that: ‘It’s all about self-respect’. This sentiment was shared by Amy who said that she received ‘personal pride’ from members visiting her garden.

## DISCUSSION

This study aimed to address a research gap in understanding the perceived health and well-being benefits of a rural garden appreciation group. Given the lack of research relating to gardening appreciation, we draw on knowledge from community gardening literature to help position the present study within the broader scholarship, and to foreground its significance.

The findings of this study indicate that the main motivation for participants to engage with CHAMPAS was to share an appreciation for gardens with a secondary motivation of viewing historical properties, and through this, build friendships and social connections within the local community. Similarly, research exploring reasons for participating in community gardening has identified that making social connections is a key motivator (Kingsley et al., 2019). However, unlike the present study, motivations for participating in community gardening also include food production, improving diet and escaping the ‘urbanity of their lives’ (Drape and Freedman, 2010; Sonti and Svendsen, 2018; Lee and Matarrita-Cascante, 2019). Potentially one of the reasons for these themes not emerging in our research is because participants were generally experienced gardeners, had their own gardens at home, and the physical action of gardening is not the remit of CHAMPAS. Supporting this observation, some research has suggested that ‘experienced’ gardeners are motivated by a social environment rather than by physical needs (Veen et al., 2016).

The building of strong social connections through CHAMPAS was a key theme as it enabled new friendships and reinforced existing ones. Participation in CHAMPAS appeared to lead to participants building broader ties to their local community through involvement with other related groups and this was perceived to positively impact participants’ well-being. This is consistent with several studies that share the observation that positive health and well-being are potentially reliant on the improved social connections associated with community gardening, specifically that these strong connections lead to increased cooperative behaviour and trust (Liamputtong and Sanchez, 2018; Shostak and Guscott, 2017; Sonti and Svendsen, 2018; Kingsley et al., 2020;). The forming of friendships is a frequent theme of past research into gardening and is often described as an element of community and social connectedness (Firth et al., 2011; Kingsley et al., 2020). Although CHAMPAS does not directly involve participants in gardening or provide a shared space for gardening (key characteristics of community gardening), it was still evident that participants made social connections through their shared interest in gardening and design, and this was a key motivation and perceived benefit from being a group member. It was interesting to note that many of the participants had a ‘web’ of connections with other community groups suggesting CHAMPAS members had a strong motivation to be involved in their community. The relationship between social connections and the well-being of CHAMPAS members was also highlighted by how the participants expressed their frustrations about the COVID-19 lockdowns and how they missed events with friends. However, it was unclear from this study whether CHAMPAS itself had any role in attracting people who have a desire to be involved in their community.

Although ‘community’ was fundamental to the participants of CHAMPAS, there did appear to be a degree of homogeneity within the group being predominantly female and older. This is consistent with the findings of a large range of research into gardening which shows a similar disproportion of gender, with groups typically having older, female participants (Shiue, 2016; Liamputtong and Sanchez, 2018; Kingsley et al., 2019; Robbins and Seibel, 2020). The group also appears to lack ethnic diversity; an issue shared by several previous community gardening studies (Glover, 2004; Firth et al., 2011; Jettner and Secret, 2020). Some describe the occurrence of this ‘ethnic homogeneity’ in gardening as a natural phenomenon that perceived racial differences are avoided because they would negatively impact the members’ sense of community (Jettner and Secret, 2020). It is suggested that some gardening groups primarily attract white, middle-class gardeners as participants, but understanding why this is still ongoing is a featured recommendation of previous research (Firth et al., 2011; Jettner and Secret, 2020). Although highlighted by members as being diverse, Colac has less diverse demographics (85.4% born in Australia) than the state of Victoria as a whole (64.9% born in Australia) (ABS, 2021). Alternatively, it should be noted that other research does suggest that gardening is a facilitator of breaking down ethnic barriers (Firth et al., 2011; Jettner and Secret, 2020).

When discussing the contribution of CHAMPAS participation to their health and well-being, well-being appeared to be more at the front of people’s minds than physical health effects. Given that a gardening appreciation group does not involve physical acts of gardening, this is not surprising. Furthermore, considering that CHAMPAS is largely middle to older-aged adults, this could be due to physically limiting age-related factors as outlined by Fielder and Marsh (2020). CHAMPAS was also perceived to reinforce mental health factors such as self-esteem and pride amongst the participants. Self-esteem has been found to be positively linked to improvements in well-being in general, as a better quality of life (de la Garza et al., 2019). For some, the source of self-esteem and pride came from self-constructed expectations about wanting to keep a good image for other members of CHAMPAS. This is further supported by Scott et al. (2014) who raise that people hold aesthetic value and a sense of achievement to gardens. This may be a unique aspect of a group such as CHAMPAS where participants visit other members’ personal gardens within their homes. These findings may be less pronounced in community gardening, which involves working together in a shared space and thus may be more communal in nature (Draper and Freedman, 2010; Veen et al., 2016; Sonti and Svendson, 2018).

In addition to the benefits derived from social connectedness, participants in CHAMPAS reported that the enjoyment of being in green spaces was fundamental to improved health. This is supported by research that suggests access to green spaces, relaxes and restores people, contributing to their well-being and the passive act of ‘viewing’ nature has therapeutic value (Ulrich, 1984; Gamble et al., 2014; Basu et al., 2018; Elsadek et al., 2020).

### Limitations

The COVID-19 pandemic and associated lockdowns during the study appeared to affect participants’ interest and involvement with CHAMPAS. With fewer activities occurring during 2020 and 2021 (particularly face-to-face events), this may have influenced participants’ perceptions of their involvement with CHAMPAS. This also made the recruitment of participants more challenging. Another limitation was that although the reflexive thematic analysis was applied in this study the findings in this paper are descriptive in nature. As Braun and Clarke (2022, p. 8) explain, thematic analysis uses ‘preexisting theory as a lens through which to interpret the data’ which this paper did not have the scope to undertake. Future studies could develop a theoretical model about people’s experiences of garden appreciation groups. Further, we acknowledge that rural areas vary across cultural, age and gender profiles, as well as across social and health indicators, and as such the experiences of people living in Colac are not directly translatable to other populations.

## CONCLUSION

This study suggests that engagement in a garden appreciation group such as CHAMPAS could potentially provide a variety of health and well-being benefits mainly through the building of social connectedness and a sense of community. This is the first study to investigate the experiences of members of a rural garden appreciation group. Given that the number of such groups in Australia and their potential benefits is unknown, a national mapping and analysis exercise in rural garden appreciation groups is recommended. As rural populations are susceptible to poorer health outcomes than those in urban areas, identifying novel health promotion initiatives that improve health and well-being may assist to reduce health inequities.

## Supplementary Material

daad010_suppl_Supplementary_Material
