# Supplementary material for: ‘Surrounding yourself with beauty’: exploring the health promotion potential of a rural garden appreciation group
Source: Health Promot Int. 2023 Feb 25;38(2):daad010. doi: 10.1093/heapro/daad010 (PMC10187782; doi:10.1093/heapro/daad010)
Supplement: daad010_suppl_Supplementary_Material [file daad010_suppl_supplementary_material.docx]

**Supplementary Material A: Interview Questions**

**Demographic data**

1. Do you live in Colac and if so for how long have you lived here?

2. What is your age?

3. What is your gender?

**General**

1. Can you tell me a little about how and why you first got involved in CHAMPAS?

a. Did you hope to gain something particularly from being involved in CHAMPAS?

2. Has your motivation for being in CHAMPAS stayed the same or changed?

3. In general, how do you feel you have benefited from being involved in CHAMPAS?

**Social:**

4. How would you describe the relationships between people in the CHAMPAS group?

a. How would you describe your relationship with this community?

5. What do you feel have been the benefits of your relationships with others involved in CHAMPAS?

6. Do you feel there is a sense of diversity within the CHAMPAS group? (age, socio-economic, gender, experience, cultural

affiliation)

7. Has your involvement with CHAMPAS shaped the way you think about this community more broadly?

**Health:**

8. Have you noticed any specific health effects from being involved in CHAMPAS?

a. And could you explain this a little more?

9. Have you noticed any wellbeing effects from being involved in CHAMPAS?

a. And could you explain this a little more?

**Improvements:**

10. Is there anything you think could be improved about CHAMPAS?

a. Regarding relationships, physical health benefits, mental health benefits
